# Syphilis

**Published:** 1902-04

**Authors:** 


SYPHILIS.—A symposium. Special contributions by L. Duncan Buckley, A. M., M. D.; Follen Cabot, Jr., M. D.; Louis A. Duhring, M. D.; Prof. Fourier, M. D.; E. Fuller, M. D.; E. B. Gleason, M. D.; W. S. Gottheil, M. D.; R. H. Greene, A. M., M. D.; N. B. Gwyn, M. D.; O. Horwitz, M. D.; E. L. Keyes, M. D.; G. F. Lydston, M. D.; D. J. McGartyh, M. D.; T. G. Morton, M. D.; Boardman Reed, M. D.; A. Robin, M. D., and J. D. Thomas, M. D. E. B. Treat & Co., New York. 1902. 122 pages. Price, $1.00 net.

All of the articles contained in this volume appeared first in the *International Medical Magazine.* Indeed, the book is really a reprint of one of the special numbers of that magazine, together with some important additions. In this remarkable collection of communications on syphilis we have the disease discussed from various standpoints by seventeen writers of reputation and ability, several of whom are numbered among the most distinguished syphilographers of this or any other country.

Questions covering many doubtful points with reference to diagnosis, drug therapy, the time when syphilitics may safely marry, whether syphilitic fathers have procreated children who have remained healthy, etc., are answered by number by Duhring, Lydston, Horwitz, Morton and Keyes. R.

---

A very timely "Treatise on Smallpox," to sell at $3.00, is announced for publication early in April by J. B. Lippincott Company. It is written by Dr. George Henry Fox, Professor of Dermatology in the College of Physicians and Surgeons, New York City, with the collaboration of Drs. S. Dana Hubbard, Sigmund Pollitzer, and John H. Huddleston, all of whom are officials of the Health Department of New York City, and have had unusual opportunities for the study and treatment of this disease during the present epidemic.

The work is to be in atlas form, similar to Fox's Photographic Atlas of Skin Diseases, published by the same house. A strong feature of the work will be its illustrations, reproduced from recent photographs, the major portion of which will be so colored as to give a very faithful representation of typical cases of variola, vaccinia, varicella, and diseases with which smallpox is liable to be confounded. These illustrations number thirty-seven, and will be grouped into ten colored plates, $9\frac{1}{2}$x$10\frac{1}{4}$ inches, and six black and white photographic plates.

The names of Dr. Fox and his associates assure the excellence of the work, in which will be described the symptoms, course of the disease, characteristic points of diagnosis, and most approved methods of treatment.

